# Creating an indicator system for the United Nations Sustainable Development Goals in communities and municipalities: Application and analysis in an Austrian case study

**DOI:** 10.1016/j.heliyon.2023.e19010

**Published:** 2023-08-09

**Authors:** Matthias Maldet, Georg Lettner, Christoph Loschan, Daniel Schwabeneder, Hans Auer

**Affiliations:** Institute of Energy Systems and Electrical Drives, Energy Economics Group (EEG), Technical University of Vienna, Vienna, Austria

**Keywords:** Sustainable development, Policy actions, Communities, Indicator establishment, Optimization model

## Abstract

Sustainability indicators should implement the United Nations Sustainable Development Goals (UN SDGs). Indicators in literature often consider large sets of actions and are thus complex in application. Therefore, this work derives energy- and resource-related SDG indicators for communities and municipalities with low complexity. Moreover, this work analyzes three different policy paths to promote SDG contribution. The policy paths consider SDG target settings and two different incentive schemes in the form of penalties and investment subsidies. The indicators and policy actions are applied in two case studies for communities and municipalities in Austria. Therefore, an optimization model that considers the case study setups, SDG targets and policy actions is developed. The modeling approach shows applicability and positive contribution to sustainable development by indicators. Moreover, the results show the applicability of the three policy paths. Implementing the target-setting path directly leads to the desired SDG targets and provides insights into the costs for target achievement. The incentive scheme paths also lead to selected targets, but they require a cost assessment of the provided incentive schemes. A combination of both incentive schemes leads to the lowest costs. However, policymakers should implement a workflow that considers all three policy paths for policy action settings.

## Highlights

• The paper introduces an SDG indicator system for communities and municipalities.
• The paper establishes three different policy paths for sustainable development.
• The method applies indicators and policies in two Austrian case studies.
• Policy setting workflows should consider a combination of the paths.
• Combining penalties and investment subsidies leads to the lowest incentive costs.

## Graphical abstract

**Figure:**
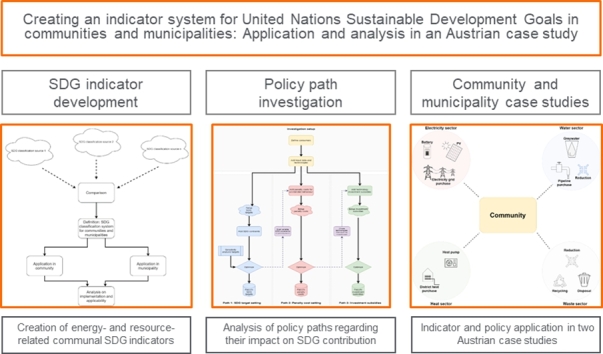

## 1 Introduction

The transition to a sustainable future is often proposed to accompany sustainable development in the present. The European Union (EU) defined sustainable development as meeting present needs while also ensuring, that future generations have no restrictions in meeting their needs. All three sustainability pillars, including social, environmental and economic pillars, must be accomplished simultaneously [1]. Therefore, the EU implemented a sustainable development strategy in 2016, that considers critical challenges such as climate change, clean energy and sustainable consumption and production. Moreover, the United Nations (UN) established a set of 17 goals for sustainable development in 2015: The United Nations Sustainable Development Goals (UN SDGs) [2]. The 17 goals provide a roadmap for the global implementation of sustainable development. The UN 2030 agenda further describes communities and local authorities as significant actors in implementing sustainable development [3]. Therefore, this work focuses on the application of UN SDG indicators in communities and municipalities, intending to provide policy incentives for decentralized sustainable development. The 17 UN SDGs consist of goals that depend on social setups and plans that can be achieved by sustainable operations and technology introduction. This work focuses on the six energy- and resource-related SDG and their interaction with community and municipality operations and investments.

**Figure 1:**
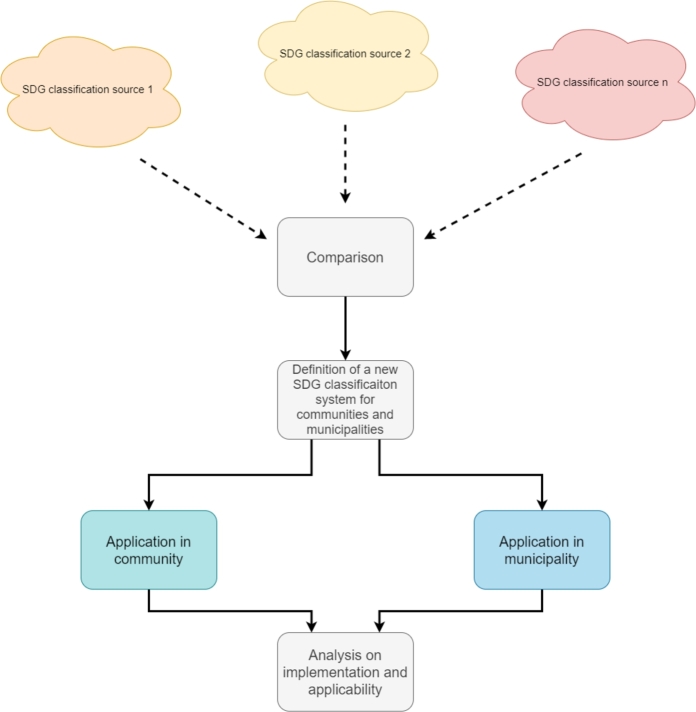
SDG indicator establishment and community/municipality application.

The UN 2030 agenda includes 169 potential actions for SDG contribution. Furthermore, much research focuses on SDG contribution and implementation. However, the majority proposes large sets of indicators and possible actions, leading to the high complexity in the application. Therefore, this work introduces an easy applicable indicator system for the energy- and resource-related SDGs. Each SDG is represented by one percentage value to make the indicators simple and comparable. The SDG indicator definition is provided based on a literature review of currently existing indicators. These are analyzed and adapted for a simple application in communities and municipalities. The developed indicators are applied to an existing community and an existing municipality in Austria by developing an optimization model. These analyses focus on the interaction between community and municipality technology investment and SDG contribution. Moreover, the studies include SDG impact assessments of different policy actions. Finally, the applicability and policy impact of the proposed SDG indicators for communities and municipalities are compared. Fig. 1 presents the workflow in the paper.

The core objectives of the analyses are to define an easily applicable SDG indicator system that provides incentives for technology investment and sustainable operation and to determine policy implications based on the indicators that can further promote sustainable development. The proposed SDG indicator system and different policy actions are applied in a community and a municipality. Therefore, an optimization model for both setups is developed to address the following research questions:

• How can the energy- and resource-related UN SDGs be established and applied in communities and municipalities?
• How do community and municipality technology portfolios and operations affect UN SDG contribution?
• Which policy actions can be established to improve UN SDG contribution?
• Which UN SDG incentive schemes are most efficient in communities and municipalities?

The paper is organized as follows: Section 2 shows existing work on sustainability in communities and municipalities. Section 3 presents a literature review on exiting SDG indicator systems and the development of an indicator system for energy- and resource-related SDGs. Section 4 shows the application of the proposed indicators in case studies for a community and a municipality. Section 5 discusses the significant results. Finally, Section 6 concludes the work by offering policy implications.

## 2 State of research

This section presents the state of research on sustainable development in communities and municipalities. Section 2.1 provides an overview of existing literature on sustainable operations in communities and municipalities. Section 2.2 presents work on sustainability indicators, and Section 2.3 focuses on work on sustainability benchmarking. Section 2.4 concludes the chapter with the progress beyond the state of research.

### 2.1 Sustainable communal operation

The UN 2030 agenda [3] proposes the importance of regional and local government involvement in sustainable development. Thus, sustainable operation in communities and municipalities is a widely discussed topic. Zahra and Badeeb [4] proposed that central governments should strengthen local governments in promoting decentralization and Ahmad and Satrovic [5] stated that decentralization can promote sustainability. Karger and Hennings [6] showed that decentralized electricity generation could positively contribute to climate protection. However, Fenton and Gustafsson [7] stated that the responsibilities and role of local actors still need clarification. Atisa et al. [8] found that local authorities lack the abilities and policies to promote a specific sustainable behavior of consumers. According to Lombardi et al. [9], the improvement in building efficiency and the promotion of tools for environmental management are fundamental actions that local authorities must address. Moreover, Ranängen et al. [10] analyzed the implementation of sustainable development by organizations, with biodiversity, climate action and freshwater as significant aspects that must be prioritized.

Technology can be an efficient means to further promote sustainable development in communities and municipalities. Kuznetsova et al. [11] examined a trend to decentralized waste treatment plants and Wang et al. [12] found green investment as an important factor in influencing sustainability. Leigh and Lee [13] examined that a transition from centralized water solutions to decentralized solutions like rainwater harvesting and greywater utilization can address urban challenges. Furthermore, Capodaglio et al. [14] introduced new development paradigms that can promote local energy and material recovery. Therefore, Thiam [15] found that support mechanisms could increase renewable energy deployment in remote areas.

In addition to investment, management of energy and resources can promote sustainable development. Thus, existing research focuses on the analysis of energy management systems. Engelken et al. [16] found that municipalities aiming for self-sufficiency can play a significant role in energy system transition. Karavas et al. [17] introduced a decentralized energy management system that is technically feasible and economically competitive. Community energy management can further improve energy management practices. Elkazaz et al. [18] introduced a hierarchical peer-to-peer management method. Hoicka et al. [19] found that energy communities can contribute to a democratic energy transition and Romero-Rubio and de Andrés Díaz [20] examined the high impact of renewable electricity owned by energy communities. Moreover, resource management methods are widely analyzed. Mesjasz-Lech [21] stated that municipalities should focus on waste reduction by creating closed cycles of materials. Jouhara et al. [22] examined substantial waste reduction in collection and transportation by introducing waste management systems. However, Periathamby [23] stated that the implementation of waste management requires impact from political authorities. Not only waste but also water must be managed in communities and municipalities. Wang and Davies [24] and Zhuang and Zhang [25] investigated different water management methods.

### 2.2 Sustainability indicators

Sustainable investment, energy and resource management can contribute to sustainable development. Bortoluzzi et al. [26] emphasized the role of multicriteria decision processes in achieving sustainable development. However, indicators must be defined to measure sustainability. Drago and Gatto [27] found that the establishment of policies is a crucial aspect of sustaining renewable energy. However, Gunnarsdottir et al. [28] examined the need for robust indicators to develop sustainable policy goals. Ameen and Mourshed [29] stated that sustainability assessment should be performed with the local context. Verma and Raghubanshi [30] introduced a multiple-step framework to define appropriate indicators. Analyses from Evans et al. [31] considered indicators, such as electricity price, greenhouse gas emission, energy and water consumption. Ngan et al. [32] found significant indicators in public acceptance and economic performance improvement and Ghenai et al. [33] introduced environmental, economic, resource, technology and social indicators as their five key indicators. Sheinbaum-Pardo et al. [34] considered governmental reasons and thus put a higher weight on economic issues than on social and environmental issues. However, Afshari et al. [35] found significant implementation challenges in potential conflicts between indicators.

Not only sustainability but also energy and resource management should be assessed by indicators. Kylili et al. [36] identified the key performance indicator approach as the most valuable assessment tool. Razmjoo et al. [37] performed an energy management assessment based on environmental impacts, renewable energy, energy access and policy. Moreover, Kourkoumpas et al. [38] introduced simple and scalable indicators, including infrastructure energy and emission reductions. Bertoldi and Mosconi [39] and Safarzadeh et al. [40] further assessed energy policy indicators concerning energy-saving promotion. According to Bertanza et al. [41], waste management indicators should consider the characteristics of the collected waste and environmental performance. Rodrigues et al. [42] proposed considering social, economic and environmental indicators in waste management. Bezerra et al. [43] stated that better coordination and problem identification are crucial for water management. Li et al. [44] examined water management practices, with low water efficiency being the most significant factor in limiting sustainable water utilization.

Special indicator systems can also be applied in communities and municipalities. According to Neves and Leal [45], indicators should be used at the beginning of the planning process to assess the current situation. However, Klemm and Wiese [46] found that not all sustainability indicators are applicable in urban energy systems. Moreover, Braulio-Gonzalo et al. [47] found that sustainability concepts vary between regions. Oliver-Solà et al. [48] analyzed municipal sustainability contribution by assessing municipal facilities' energy consumption and greenhouse gas emissions. Alonso et al. [49] proposed that cities must implement a circular economy as a sustainability indicator. Caldas et al. [50] aggregated indicators for local government sustainability performance assessment. Furthermore, Teixeira et al. [51] developed indicators for municipal water management, establishing four categories, namely, environmental, social, technical and governance categories.

### 2.3 Sustainability benchmarking

The concept of sustainability indicators can be extended to sustainability benchmarking programs by comparing consumers' sustainable development with sustainability indicators. According to Chung [52], benchmarking might encourage poorly performing consumers to improve their performance. Moreover, Roth and Rajagopal [53] stated that robust benchmarking programs might improve resource allocation for energy efficiency programs. Dubey et al. [54] emphasized that sustainability benchmarking is becoming increasingly crucial in industry. Moreover, Şiir Kılkış [55] stated that decision-makers can use benchmarking results as a planning tool. Trigaux et al. [56] developed benchmarking recommendations for the building sector with a transparent and user-friendly system being a significant aspect. Furthermore, Lazar and Chithra [57] implemented benchmarking systems for worst and best building performances and Xuchao et al. [58] developed a regression-based benchmarking model. Ding and Liu [59] compared three benchmarking approaches, and they propose that policymakers should apply multiple benchmarking tools. Welling and Ryding [60] identified life cycle assessment as an effective method for environmental impact measurement and Hollberg et al. [61] found that life cycle assessment-based benchmarks have been used as certification systems on the building level.

Many existing programs already implement benchmarking for technologies, communities or municipalities. The EU taxonomy classifies sustainability contribution and investment by developing technical screening criteria [62]. Therefore, the taxonomy provides sustainability indicators in the form of the technical screening criteria. The Austrian energy certificate is an energy benchmarking tool that classifies building energy efficiency. It is thus a simple benchmarking tool for buildings [63]. The certificate is derived from EU energy certificates [64]. A similar approach is implemented in the EU energy labels. These labels classify the energy efficiency of products and devices [65]. Furthermore, community and municipality benchmarking programs are implemented in different EU countries. The “e5” program in Austria supports municipalities in sustainable operation and rewards sustainable behavior with a five-star certification benchmarking system. Moreover, the program proposes potential actions for a transition to a higher certification [66], [67], [68]. The network of energy cities is a similar program that supports concept exchange between associated cities and municipalities [69].

### 2.4 Novelties and progress beyond the state of research

Sustainable development in communities and municipalities is a widely researched topic. Existing work focuses strongly on sustainability indicators and benchmarking programs. However, sustainability indicators are often extensive and complex, thus leading to a high level of complexity in community or municipality applications. Many developed sustainability indicators also do not directly refer to the UN SDGs. Moreover, the policy impacts of SDG indicator applications are often not analyzed. This work addresses the complexity of UN SDG indicator systems by deriving a new, easy applicable indicator system. The implementation and applicability of the proposed indicator system are tested in community and municipality case studies. Furthermore, this research provides policy implications based on the proposed indicator system and case studies.

The novelties and progress beyond state of research include the following aspects:

i) This research develops a UN SDG indicator system for communities and municipalities that is transparent and easily applicable.
ii) The method combines the proposed indicator system with an optimization modeling approach.
iii) This research applies and analyzes the proposed system in case studies for a community and a municipality.
iv) The analyses assess different policy actions for UN SDG contribution improvement.

## 3 Sustainable development goals: community and municipality classification

This section introduces a review of SDG contribution and indicator systems. Section 3.1 presents the state of research on SDG application in communities and municipalities. Section 3.2 provides an overview of existing SDG indicator systems. Finally, Section 3.3 proposes a new SDG indicator system applicable to communities and municipalities. The nomenclature in Table 6 describes the mathematical symbols used in the indicator definition.

### 3.1 UN SDGs in communities and municipalities

Section 2.2 introduced existing sustainability indicators, with some applicable in communities and municipalities. However, most of those indicators do not directly refer to the UN SDGs. As this work focuses on an appropriate SDG indicator system with a joint base, this section analyzes literature that focuses explicitly on SDG contribution.

Szetey et al. [70] stated that local communities must focus on local relevant indicators for SDG achievement. Therefore, they introduced pathways, including people, property and planet. Quiroz-Niño and Ángeles Murga-Menoyo [71] found that training sustainability competencies is mandatory for achieving SDGs. Bardal et al. [72] stated the importance of local authorities in goal implementation. According to Krantz and Gustafsson [73], municipality involvement in SDG achievement is crucial because of the municipal range of responsibilities. This was further emphasized by Teixeira et al. [74], as they proposed that municipalities become leaders in engaging SDG contribution. Salvia et al. [75] further highlighted the importance of local governments in resource management. However, Ślusarczyk and Grondys [76] underlined that municipalities should belong to economic zones to achieve sustainable development. Moreover, Fenton et al. [77] stated that municipal energy strategy development depends on the choice of communicative approaches. Han et al. [78] found that policymaking depends on indicators' importance. However, Meyar-Naimi and Vaez-Zadeh [79] examined the importance of considering national visions in policymaking.

Bain et al. [80] stated that most countries could generate estimates for SDG6 (clean water and sanitation), but SDG12 (responsible consumption and production) was less reported. Therefore, Razali et al. [81] suggested fostering household waste separation behavior and Pujara et al. [82] emphasized the importance of minimizing waste landfilling. Moreover, Santika et al. [83] examined energy efficiency measures as an essential aspect of contributing to SDG7 (clean and affordable energy). Dioha and Emodi [84] examined significant energy demand reductions by providing modern energy access. Fan et al. [85] found energy consumption dependency on socioeconomic factors and physical conditions. However, Fraisl et al. [86] stated that information on citizen contribution to SDG indicators must be included.

### 3.2 Quantification of the UN SDG

Focusing on SDG contribution is common to the introduced analyses. However, no clear indicators or targets are defined. Therefore, this section focuses on SDG quantification. The primary focus is set on energy- and resource-related SDG.

According to Hák et al. [87], indicators should consider policy relevance, a link to the target and applicability. Therefore, Miola and Schiltz [88] analyzed three different indicator methods, namely, mean evaluation, distance measure and progress determination. Swain and Yang-Wallentin [89] introduced indicator equations to identify SDG contribution. Costanza et al. [90] linked the SDG with a defined well-being index and Kubiszewski et al. [91] applied linear regression to determine indicators. Furthermore, Mischen et al. [92] performed a community assessment to define indicators.

Even though much literature focuses on SDG quantification, the design of an SDG indicator system requires reference to widely applicable goals and norms. The UN SDGs propose 17 goals with 169 practical actions to reach these goals [2]. The energy- and resource-related goals are SDG6 (clean water and sanitation), SDG7 (clean and affordable energy), SDG11 (sustainable cities and communities), SDG12 (responsible consumption and production), and SDG13 (climate action). Moreover, SDG1 (no poverty) must be considered to keep the financial load on consumers at an acceptable level. The UN recognized regional economic integration in their 2030 agenda [3]. They proposed that regional levels can provide valuable opportunities for peer learning. Moreover, the 2030 agenda focuses on the application of sustainable actions for each goal. However, the number of actions might result in the high complexity in the application.

The ISO norm 37120 for sustainable cities and communities [93] introduces core indicators and supportive indicators to measure sustainable development. The norm categories energy, environment, finance, solid waste, wastewater and water and sanitation can be relevant for energy- and resource-related SDGs. According to Moschen et al. [94], ISO 37120 does not specify ideal actions for sustainable development regarding the UN SDGs. Therefore, the norm should be seen as an additional sustainability indicator rather than a direct recommendation for sustainable development according to the UN SDGs.

Furthermore, the Organization for Economic Co-operation and Development (OECD) proposes an action plan for countries to define actions leading to UN SDG contributions [95]. Therefore, they introduce a set of targets for each UN SDG [96]. However, the targets are defined for national policies and are therefore more suitable for national UN SDG indication. Jossin and Peters [97] introduced an SDG indicator system that is applicable in municipalities. They raised 120 indicators, that cover all SDGs. However, similar to the UN SDG actions [3], the proposed actions by Jossin and Peters [97] might lead to high complexity.

Table 1, Table 2 present an overview of the energy- and resource-related SDG indicators that are proposed by the [3], [93], [96] and Jossin and Peters [97]. In the comparison, identified relevant indicators in the proposed systems are considered.

**Table 1:** Comparison of different proposed sustainable development indicators, SDG 1, 6 and 7.

SDG	UN indicators	ISO 37120	OECD	Jossin and Peters
1: No poverty	Proportion of population below poverty level	Percentage of population living in poverty	Poverty rate	Gini coefficient
6: Clean water and sanitation	Degree of integrated water resources management, proportion of wastewater flows	Percentage of population with potable water supply and wastewater treatment	Share of population without wastewater collection	Drinking water consumption, percentage of treated wastewater
7: Affordable and clean energy	Renewable energy share in final energy consumption	Percentage of energy derived from renewable sources	Renewable electricity share in electricity generation	Renewable energy in energy consumption, municipal investment in development

**Table 2:** Comparison of different proposed sustainable development indicators, SDG 11, 12 and 13.

SDG	UN indicators	ISO 37120	OECD	Jossin and Peters
11: Sustainable cities and communities	Proportion of solid waste managed	ISO 37120 is established for sustainable city and community indication	Municipal waste generated	Combination of multiple categories including energy and resources
12: Responsible consumption and production	National recycling rate, installed renewable energy generation capacities	Number of recycled waste, reduced waste or landfilled waste	Recycling rate of municipal waste	Drinking water consumption, energy consumption, waste generation, recycling rate
13: Climate action	Total GHG emissions per year	Total GHG emissions per capita	Production based CO_2_ emissions	CO_2_ emissions in private household and municipal facilities

### 3.3 Introduction od the UN SDG indicator system

The proposed indicator systems in Table 1, Table 2 show differences in particular SDG targets. SDG11 can be widely interpreted, which can be seen as the sources considered waste management and multiple other energy- and resource-related indicators. SDG12 is also not strictly limited to waste by all sources. Moreover, different indicators for the different SDGs are not comparable to each other, making an overall comparable indicator system hardly applicable. Therefore, this paper proposes an adaptation of the proposed indicators in Table 1, Table 2 to a newly-defined SDG indicator system. The indicator system is designed to be applicable in sustainable communities and cities, especially for application in Austria. However, different indicators for both communities and municipalities are implemented because of their different scopes. Furthermore, to make the indicators comparable, each indicator is defined as a percentage value. A higher value indicates better contribution to a particular SDG. The goal is to implement an easily applicable SDG indicator system that reflects sustainable development contribution and provides appropriate incentives.

SDG1 (no poverty) $i^{\mathrm{nopovert}y_{\mathrm{SDG1}}}$ is equally indicated in communities and municipalities. It is defined as the cost reduction that can be achieved by sustainable technology implementation. For providing an SDG1 indicator, total cost improvement $c^{\mathrm{tot}}$ compared with business-as-usual costs $c^{\mathrm{tot},\mathrm{BaU}}$, for the same community or municipality without sustainable technology installation are considered. This benchmark is required to provide a percentage value for the indicator. The target is described in Equation (1).

(1) $$\begin{array}{c} i^{\mathrm{nopovert}y_{\mathrm{SDG1}}}=\frac{c^{\mathrm{tot},\mathrm{BaU}}-c^{\mathrm{tot}}}{c^{\mathrm{tot},\mathrm{BaU}}} \end{array}$$

SDG6 (clean water and sanitation) $i_{com}^{\mathrm{cleanwate}r_{\mathrm{SDG6}}}$ is indicated differently in communities and municipalities. Communities (see Equation (2)) consider the amount of reduced $v_{water}^{\mathrm{reduced}}$ and reused water in the form of greywater $v_{water,com}^{\mathrm{greywater}}$ in relation to the total water demand $D_{water,com}$.

(2) $$\begin{array}{c} i_{com}^{\mathrm{cleanwate}r_{\mathrm{SDG6}}}=\frac{v_{water,com}^{\mathrm{reduced}}+v_{water,com}^{\mathrm{greywater}}}{D_{water,com}} \end{array}$$

The SDG6 indicator for municipalities $i_{mun}^{\mathrm{cleanwate}r_{\mathrm{SDG6}}}$ extends the enumerator to recovered water from sewage treatment $v_{water,mun}^{\mathrm{recovered}}$, which can be used for water demand coverage. Equation (3) presents the indicator.

(3) $$\begin{array}{c} i_{mun}^{\mathrm{cleanwate}r_{\mathrm{SDG6}}}=\frac{v_{water,mun}^{\mathrm{reduced}}+v_{water,mun}^{\mathrm{greywater}}+v_{water,mun}^{\mathrm{recovered}}}{D_{water,mun}} \end{array}$$

The SDG7 (clean and affordable energy) indicator is also implemented differently in communities and municipalities. The indicator $i_{com}^{\mathrm{cleanenerg}y_{\mathrm{SDG7}}}$ considers the share of renewable energy procurement $q_{el,com}^{\mathrm{ren}}$, in relation to the total energy procurement $q_{el,com}^{\mathrm{tot}}$. Energy procurement includes PV generation $q_{el,com}^{\mathrm{PV}}$, electricity grid procurement $q_{el,com}^{\mathrm{elgrid}}$, heat pump heat generation $q_{heat,com}^{\mathrm{HP}}$ and district heat procurement $q_{heat,com}^{\mathrm{dhgrid}}$. For the renewable share of grid procurement, the percentage of renewable energy in the electricity $F_{elgrid}^{\mathrm{ren}}$ and heat mix $F_{dhgrid}^{\mathrm{ren}}$ are considered. Moreover, electricity feed-in $q_{el,com}^{\mathrm{feedin}}$ is subtracted in the enumerator to consider efficient energy utilization and to facilitate the local use of renewable energy. Equations (4) to (6) present the indicator for communities.

(4) $$\begin{array}{cc} q_{el,com}^{\mathrm{ren}}=q_{el,com}^{\mathrm{PV}} & +F_{elgrid}^{\mathrm{ren}}\cdot q_{el,com}^{\mathrm{elgrid}}+q_{heat,com}^{\mathrm{HP}}\\ & +F_{dhgrid}^{\mathrm{ren}}\cdot q_{heat,com}^{\mathrm{dhgrid}}-q_{el,com}^{\mathrm{feedin}} \end{array}$$

(5) $$\begin{array}{c} q_{el,com}^{\mathrm{tot}}=q_{el,com}^{\mathrm{PV}}+q_{el,com}^{\mathrm{elgrid}}+q_{heat,com}^{\mathrm{HP}}+q_{heat,com}^{\mathrm{dhgrid}} \end{array}$$

(6) $$\begin{array}{c} i_{com}^{\mathrm{cleanenerg}y_{\mathrm{SDG7}}}=\frac{q_{el,com}^{\mathrm{ren}}}{q_{el,com}^{\mathrm{tot}}} \end{array}$$

Municipal SDG7 indicators $i_{mun}^{\mathrm{cleanenerg}y_{\mathrm{SDG7}}}$ additionally consider recovered electricity $q_{el,mun}^{\mathrm{wastecomb}}$ and heat $q_{el,mun}^{\mathrm{wastecomb}}$ from waste incineration (see Equations (7) to (9)). However, in the enumerator, only the biogenic share of waste $F_{waste}^{\mathrm{biogene}}$ is counted as renewable. Furthermore, exhaust heat $q_{heat,mun}^{\mathrm{exhaust}}$ is considered in the enumerator to efficiently utilize locally generated heat in municipalities.

(7) $$\begin{array}{cc} q_{el,mun}^{\mathrm{ren}}=q_{el,mun}^{\mathrm{PV}} & +F_{elgrid}^{\mathrm{ren}}\cdot q_{el,mun}^{\mathrm{elgrid}}+q_{heat,mun}^{\mathrm{HP}}\\ & +F_{dhgrid}^{\mathrm{ren}}\cdot q_{heat,mun}^{\mathrm{dhgrid}}-q_{el,mun}^{\mathrm{feedin}}\\ & +F_{waste}^{\mathrm{biogene}}\cdot (q_{el,mun}^{\mathrm{wastecomb}}+q_{heat,mun}^{\mathrm{wastecomb}})-q_{heat,mun}^{\mathrm{exhaust}} \end{array}$$

(8) $$\begin{array}{cc} q_{el,mun}^{\mathrm{tot}}=q_{el,mun}^{\mathrm{PV}} & +q_{el,mun}^{\mathrm{elgrid}}+q_{heat,mun}^{\mathrm{HP}}+q_{heat,mun}^{\mathrm{dhgrid}}\\ & +q_{el,mun}^{\mathrm{wastecomb}}+q_{heat,mun}^{\mathrm{wastecomb}} \end{array}$$

(9) $$\begin{array}{c} i_{mun}^{\mathrm{cleanenerg}y_{\mathrm{SDG7}}}=\frac{q_{el,mun}^{\mathrm{ren}}}{q_{el,mun}^{\mathrm{tot}}} \end{array}$$

SDG11 is not represented by a single indicator, but rather considers a combination of all other energy- and resource-related indicators. The concept of communities and municipalities applying an SDG indicator system is automatically a contribution to SDG11. Each indicator is weighted by its contributions, compared to the overall contribution (see Equation (10)). All weighted indicator contributions sum up to , as presented in Equation (11).

(10) $$\begin{array}{c} i_{new}^{\mathrm{SDG},k}=\frac{i_{old}^{\mathrm{SDG},k}}{\sum_{j\in SDGs}i_{old}^{\mathrm{SDG},j}}\;\forall k\in \mathrm{SDG} \end{array}$$

(11) $$\begin{array}{c} \sum_{k\in \mathrm{SDG}}i_{new}^{\mathrm{SDG},k}=100\text{\%} \end{array}$$

SDG12 is indicated equally in communities and municipalities by $i^{\mathrm{conspro}d_{\mathrm{SDG12}}}$. The indicator considers the ratio of reduced and recycled waste to the total accruing waste, as presented in Equation (12).

(12) $$\begin{array}{c} i^{\mathrm{conspro}d_{\mathrm{SDG12}}}=\frac{m_{waste}^{\mathrm{recycled}}+m_{waste}^{\mathrm{reduced}}}{M_{waste}^{\mathrm{total}}} \end{array}$$

Finally, the SDG13 indicator $i^{\mathrm{climateactionSDG13}}$ considers the emissions $em^{\mathrm{tot}}$ compared with the BaU scenario emissions $em^{\mathrm{tot},\mathrm{BaU}}$ of the community or municipality, similar to SDG1 (see Equation (13)).

(13) $$\begin{array}{c} i^{\mathrm{climateactio}n_{\mathrm{SDG13}}}=\frac{em^{\mathrm{tot},\mathrm{BaU}}-em^{\mathrm{tot}}}{em^{\mathrm{tot},\mathrm{BaU}}} \end{array}$$

The paper considers the proposed indicators for further analyses and discussions. Table 3 summarizes the developed SDG indicators.

**Table 3:** Proposed SDG contribution indicators for communities and municipalities.

SDG	Community indicator	Municipality indicator
1: No poverty	Community cost reduction compared to BaU in % (Equation (1))	Municipality cost reduction compared to BaU in % (Equation (1))
6: Clean water and sanitation	Percentage of reduced water and reused greywater in relation to community water demand (Equation (2))	Percentage of reduced water, reused greywater and recovered water from sewage treatment in relation to municipality water demand (Equation (3))
7: Affordable and clean energy	Community share of renewable energy generation, excluding fed-in energy, in % (Equation (6))	Municipality share of renewable energy generation, including the biogenic share of waste incineration and excluding fed-in energy, in % (Equation (9))
11: Sustainable cities and communities	Combination impact of other SDGs in communities	Combination impact of other SDGs in municipalities
12: Responsible consumption and production	Community share of reduced and recycled waste to accruing waste (Equation (12))	Municipality share of reduced and recycled waste to accruing waste (Equation (12))
13: Climate action	Community emission reduction compared to BaU in % (Equation (13))	Municipality emission reduction compared to BaU in % (Equation (13))

## 4 Community and municipality analyses

This section applies the proposed SDG indicator system in case studies for an existing community and municipality. Section 4.1 presents the case study methodology and Sections 4.2 and 4.3 introduce the setups and results of the community and municipality analyses.

### 4.1 Case study, materials and method

The method is applied in the case studies for the community and municipality. The case study parameters are summarized in the Appendix. Both, communities and municipalities, are analyzed by optimization models, representing the energy- and resource-related operations and investments in the systems. Therefore, the optimization modeling framework “Resource Utilization in Sector Coupling” (RUTIS) [98] is extended to particular SDG target achievement functionalities. A validation of the model is also presented in [99]. The model implements a cost minimization, represented in Equation (14).

(14) $$min(z)=min(c^{\mathrm{tot}})=min(c^{\mathrm{procurement}}+c^{\mathrm{operational}}+c^{\mathrm{invest}})$$

Procurement costs $c^{\mathrm{procurement}}$ represent the costs for external energy or resource procurement and operational costs $c^{\mathrm{operational}}$ represent costs for technology operation. Both are multiplied by the amount of procured or operated energy and resources. Investment costs $c^{\mathrm{invest}}$ are considered with annuities $\alpha_{l}$, multiplied by the installed technology capacity $x_{l}$, whereas the capacity is determined by the optimization. Annuities consider the weighted average cost of capital WACC and the amortization period of the technologies $N_{l}$ Equations (15) and (16) present the model implementation.

(15) $$\alpha_{l}=\frac{{\left(1+WACC\right)}^{N_{l}}\cdot WACC}{{\left(1+WACC\right)}^{N_{l}}-1}\;\forall l\in T$$

(16) $$c_{l}^{\mathrm{invest}}=\alpha_{l}\cdot x_{l}\cdot C_{l}^{\mathrm{invest}}\;\forall l\in T$$

Basic model constraints include conversion relations, technology limitation, balance rules for all sectors and storage equations. Detailed RUTIS model constraint equations are presented in [99].

**Figure 2:**
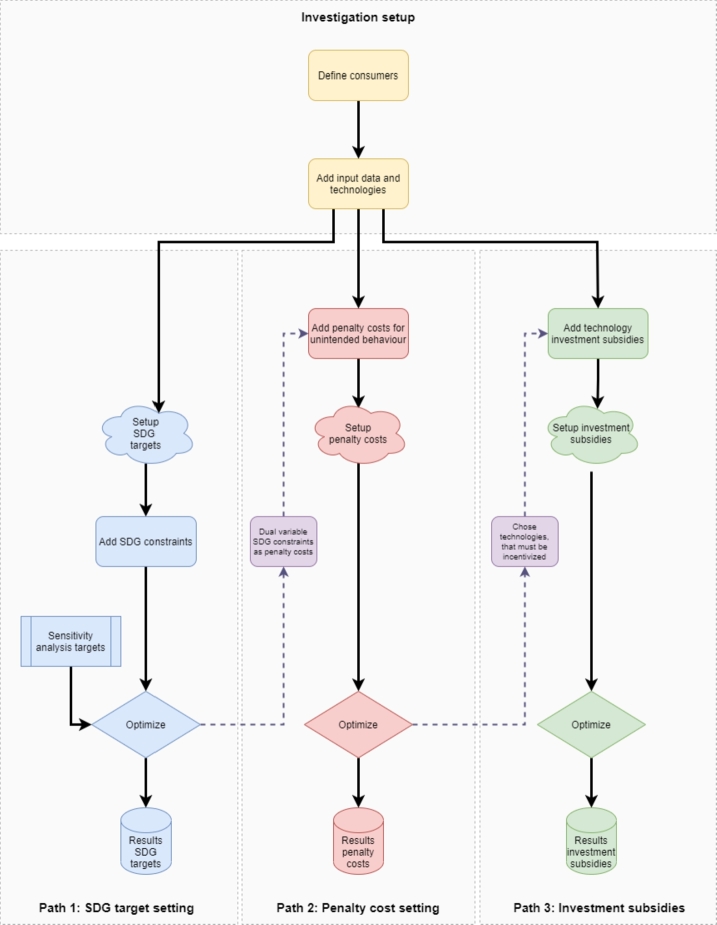
Case study policy paths.

The case study applies three different policy paths, where various policy actions are applied in the community and municipality. All three policies aim to improve sustainable development and contribution to the UN SDG, whereas the particular policy actions differ depending on the path. Fig. 2 presents the workflow of the policy paths.

Path 1 represents strict target setting, where particular SDG contribution targets must be strictly achieved by technology installation and sustainable behavior. Analyses in Path 1 include sensitivity analyses on SDG target achievements, whereas changes in technology portfolios and total costs are examined. Sensitivity analysis for a simultaneous increase in all SDG targets leads to different limits for different SDGs. Thus, if an SDG target is at its limit, the goal is set to the maximum possible value for the following sensitivity values. The SDG targets are implemented as additional model constraints. The dual variables of the constraints are extracted to analyze the impact and costs of the limitations, particularly target achievement. The constraints are derived from the indicator system presented in Table 3. However, it must be considered that SDG6 and SDG7 indicators are implemented differently in communities and municipalities. Equations (17) and (18) present the model implementation of SDG6 for communities and municipalities. Equations (19) and (20) present the model constraints for SDG7 while Equation (21) presents the implementation of SDG12 in the model. Finally, Equation (22) implements SDG13 in the model. *TG* describes a predefined SDG target and *λ* represents the dual variables. Terms for water or waste reduction are not implemented in all analyses and can be considered additional sensitivities.

(17) $$\begin{array}{c} \frac{v_{water,com}^{\mathrm{reduced}}+v_{water,com}^{\mathrm{greywater}}}{D_{water,com}}\geq TG_{com}^{\mathrm{cleanwate}r_{\mathrm{SDG6}}}\;:\lambda_{com}^{\mathrm{cleanwate}r_{\mathrm{SDG6}}} \end{array}$$

(18) $$\begin{array}{c} \frac{v_{water,mun}^{\mathrm{reduced}}+v_{water,mun}^{\mathrm{greywater}}+v_{water,mun}^{\mathrm{recovered}}}{D_{water,mun}}\geq TG_{mun}^{\mathrm{cleanwate}r_{\mathrm{SDG6}}}\;:\lambda_{mun}^{\mathrm{cleanwate}r_{\mathrm{SDG6}}} \end{array}$$

(19) $$\begin{array}{c} \frac{q_{el,com}^{\mathrm{ren}}}{q_{el,com}^{\mathrm{tot}}}\geq TG_{com}^{\mathrm{cleanenerg}y_{\mathrm{SDG7}}}\;:\lambda_{com}^{\mathrm{cleanenerg}y_{\mathrm{SDG7}}} \end{array}$$

(20) $$\begin{array}{c} \frac{q_{el,mun}^{\mathrm{ren}}}{q_{el,mun}^{\mathrm{tot}}}\geq TG_{mun}^{\mathrm{cleanenerg}y_{\mathrm{SDG7}}}\;:\lambda_{mun}^{\mathrm{cleanenerg}y_{\mathrm{SDG7}}} \end{array}$$

(21) $$\begin{array}{c} \frac{m_{waste}^{\mathrm{recycled}}+m_{waste}^{\mathrm{reduced}}}{M_{waste}^{\mathrm{total}}}\geq TG^{\mathrm{conspro}d_{\mathrm{SDG12}}}\;:\lambda^{\mathrm{conspro}d_{\mathrm{SDG12}}} \end{array}$$

(22) $$\begin{array}{c} \frac{em^{\mathrm{tot},\mathrm{BaU}}-em^{\mathrm{tot}}}{em^{\mathrm{tot},\mathrm{BaU}}}\geq TG^{\mathrm{climateactio}n_{\mathrm{SDG13}}}\;:\lambda^{\mathrm{climateactio}n_{\mathrm{SDG13}}} \end{array}$$

The policy actions in Path 2 use the dual variables to create penalties for actions leading to lower SDG targets. Moreover, technologies that are installed to avoid penalties can be identified in Path 2. Path 3 considers policy actions in the form of investment subsidies for the recognized technologies. Additional costs in both paths, namely, penalty costs for consumers in Path 2 and subsidy costs for the funding agency in Path 3, are considered within the system boundaries, as subsidies are paid by consumers' taxes. Therefore, for the evaluation, penalties and subsidies are considered cost loads for the consumers. Finally, the aim of the analyses is to compare the three paths in the community and municipality setups.

### 4.2 Community analyses

The community analyses in this section present the investigation setup in Section 4.2.1, the results of the studies for target setting (Path 1) in Section 4.2.2 and the results for incentive schemes (Paths 2 and 3) in Section 4.2.3. Additional results of the analyses are presented in the Appendix.

#### 4.2.1 Community investigation setup

**Figure 3:**
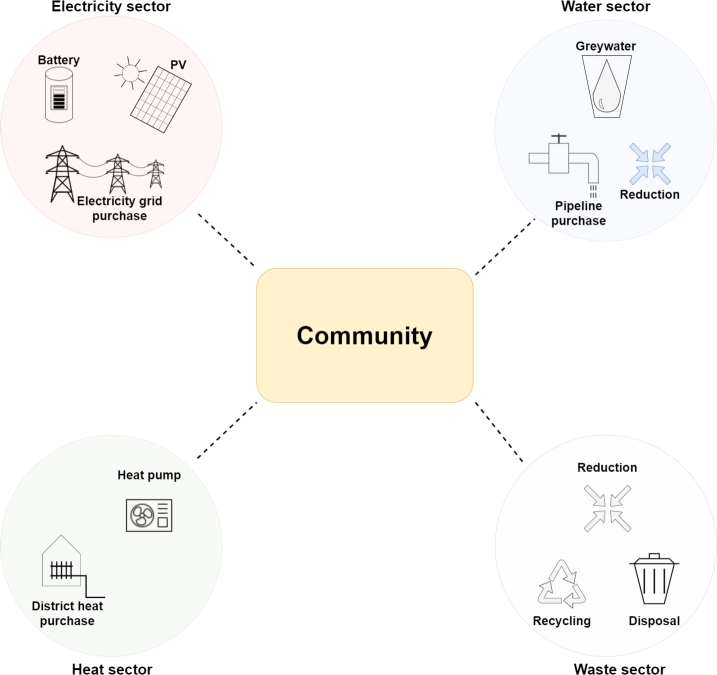
Case study setup for community analyses.

The sustainable community “Gemeinschaftlich Wohnen die Zukunft” (GeWoZu) [100], consisting of 12 households, is considered for the community analyses. Consumers in the community are aggregated. Fig. 3 presents the investigation setup in the community.

The model performs investment decisions in PV and batteries in the electricity sector. Excess electricity can be fed into the grid and the remaining demand is covered by electricity grid procurement. The heat sector considers investment decisions in heat pumps and district heat connections, whereas heat can be procured from an external heat source for the latter. Potable water is usually procured from pipelines. However, investment decisions in separate greywater systems and sensitivities for water reduction are additionally considered for the water sector. The waste sector includes competition between waste disposal and recycling, which are implemented with different costs. Similar to the water sector, waste reduction is considered as sensitivity.

#### 4.2.2 Politically driven goal achievement in communities

This section presents the results of the analyses for SDG target policy actions (Path 1). First, single SDG contribution target sensitivity analyses are presented, followed by a complete sensitivity analysis on all SDGs.

**Figure 4:**
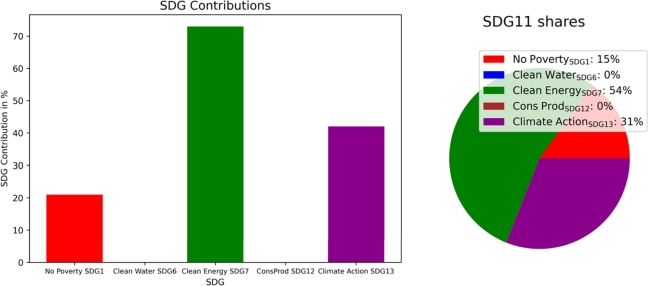
Community contribution to SDGs (left) and share of single SDG contributions to SDG11 (sustainable cities and communities) (right).

Without constraints, the total costs can be reduced by  compared to the business-as-usual (BaU) setup because of the financial benefits due to clean technology installation. SDG6 (clean water and sanitation) and SDG12 (responsible consumption and production) are not targeted, whereas SDG7 (clean and affordable energy) and SDG13 (climate action) reach  and , respectively. Thus, even without particular policy actions, clean technology installation leads to increased SDG7 contributions and to total cost reductions. Fig. 4 presents the target achievement for the energy- and resource-related SDGs without target achievement constraints. Additionally, it shows the contribution of SDG11 (sustainable cities and communities).

**Figure 5:**
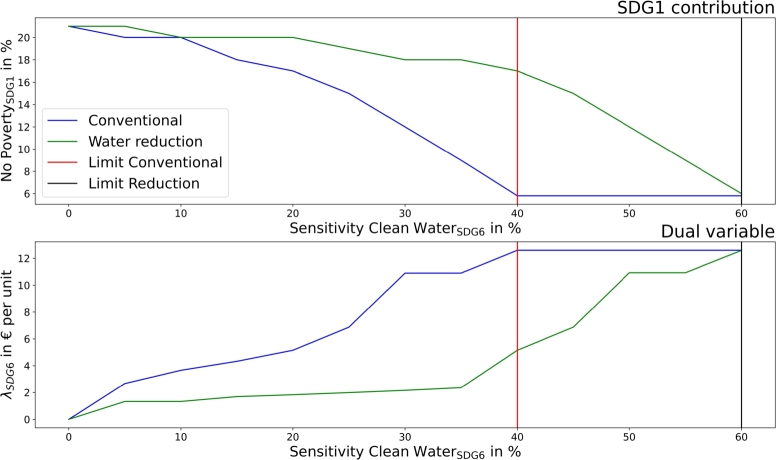
SDG1 - no poverty (top) and the dual variable of SDG6 (clean water and sanitation) in dependency of SDG6 contribution targets in the community.

Fig. 5 presents the impact of the sensitivity analysis of SDG6 on the total costs (represented with SDG1) and on the dual variables of SDG6, comparing a conventional setup without options for water reductions and the additional consideration of such.

A community can only achieve clean water and sanitation (SDG6) improvement by installing greywater systems. However, as the share of greywater in sewage is limited, SDG6 has its limit at . The high costs for target achievement can be seen in SDG1 decreases and the dual variables. Water reduction of  leads to a linear shift of the limit and dual variable and to lower costs at higher targets.

**Figure 6:**
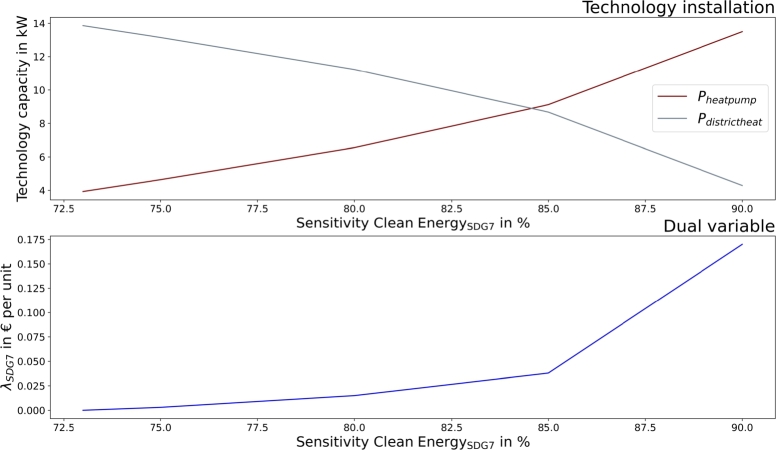
Community heat technology installation (top) and the dual variable of SDG7 (clean and affordable energy) in dependency of SDG7 contribution targets.

SDG7 (clean and affordable energy) targets can be achieved by favoring heat pump installation to district heat connection, as presented in Fig. 6. Contributions up to  can be achieved. Moreover, batteries are installed to promote local renewable energy use and to prevent electricity grid feedin. The dual variables define the costs for additional target achievement. They are low compared to SDG 6. However, target increase from  to  leads to a sharp increase in the dual variables.

**Figure 7:**
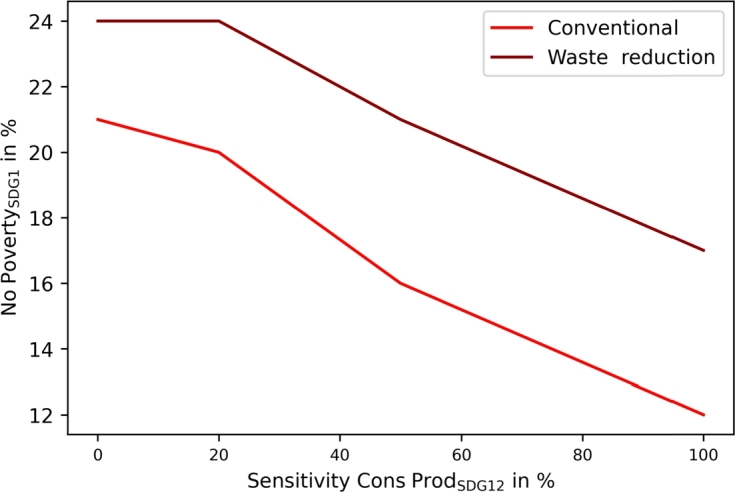
Community sensitivity analysis for SDG12 (responsible consumption and production) with and without waste reduction.

Fig. 7 presents the sensitivity analysis of SDG12 (responsible consumption and production), where additional recycling leads to higher costs. The increase can be lowered by promoting waste reduction.

**Figure 8:**
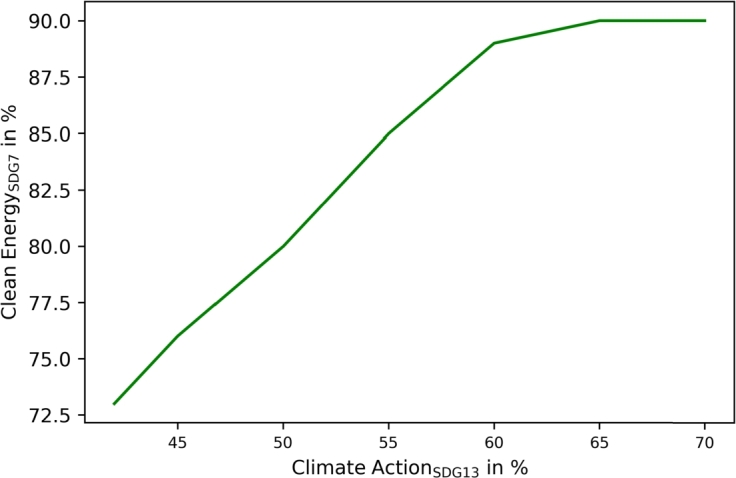
Correlation between SDG13 (climate action) and SDG7 (clean and affordable energy) in communities.

Finally, Fig. 8 shows a direct correlation between SDG7 and SDG13 (climate action). However, at SDG13 contribution targets over , only SDG7 slightly increases as resource-related operations such as greywater installation and waste recycling are increasingly implemented, which do not directly contribute to SDG7.

The simultaneous sensitivity analysis results show that different SDG targets become active at different limits. According to the Karush-Kuhn-Tucker (KKT) conditions, a constraint and an SDG target become active if the respective dual variables are not equal to zero. SDG6 and SDG12 are the first active constraints at , followed by SDG13 at . Owing the correlation with SDG13, SDG7 is the last functional constraint at . Limits are similar to the single-goal sensitivity analyses, with SDG6 limit at , SDG13 at , and SDG7 at . As no limit for waste recycling was assumed, the constraint is not limited in target setting. Detailed results of the simultaneous sensitivity analysis are presented in the Appendix.

#### 4.2.3 Politically driven incentive schemes in communities

This section presents the results of the incentive schemes in the form of penalties (Path 2) and investment subsidies (Path 3). Detailed results on Path 2 and Path 3 analyses are presented in the Appendix. Incentive schemes in Paths 2 and 3 both lead to the same SDG contributions as predefined targets in Path 1. However, the incentive schemes differ in their impact on the total costs and in cost-requirement for penalizing or providing incentives. Table 4 presents a comparison between different incentive schemes.

**Table 4:** Comparison of policy paths 2 and 3 regarding incentive cost volume, total community costs and cost increases in the community.

Policy	Incentive	Incentive costs in €	Total community costs in €	Cost increase in %
No incentives	-	0	18723	0
Sewage disposal penalty		10064	20701	10.57
Greywater incentive		2020	20464	9.30
District heat procurement penalty		194	18979	1.37
Heat pump subsidies		506	18875	0.81
Waste disposal penalties		0	20895	11.60
Waste recycling subsidies		2228	20895	11.60
CO_2_ price	_CO2_	8001	20413	9.02
CO_2_ price	_CO2_	697	18771	0.26
Combination penalties	-	10650	23184	23.83
Combination subsidies	-	5311	23811	27.17
Combination half subsidies, half penalties	-	3011	23161	23.70

SDG6 improvement leads to the highest incentive costs. Greywater installation requires  of incentive costs of sewage disposal penalties. The relations differ in SDG7, where penalties for district heat procurement lead to lower incentive costs than heat pump investment subsidies. SDG12 incentives are independent of the implementation. Waste disposal penalties automatically prevent disposal, leading to higher costs for consumers. SDG13 improvement requires comparably high costs. Considering a combination of incentive schemes leads to higher incentive costs for penalties compared with investment subsidies. However, a combination of both, penalties and subsidies, leads to the lowest incentive costs.

### 4.3 Municipality analyses

This section presents the municipality setup in Section 4.3.1, the SDG target setting of the municipality in 4.3.2, and the incentive schemes for the municipality in Section 4.3.3.

#### 4.3.1 Municipality investigation setup

**Figure 9:**
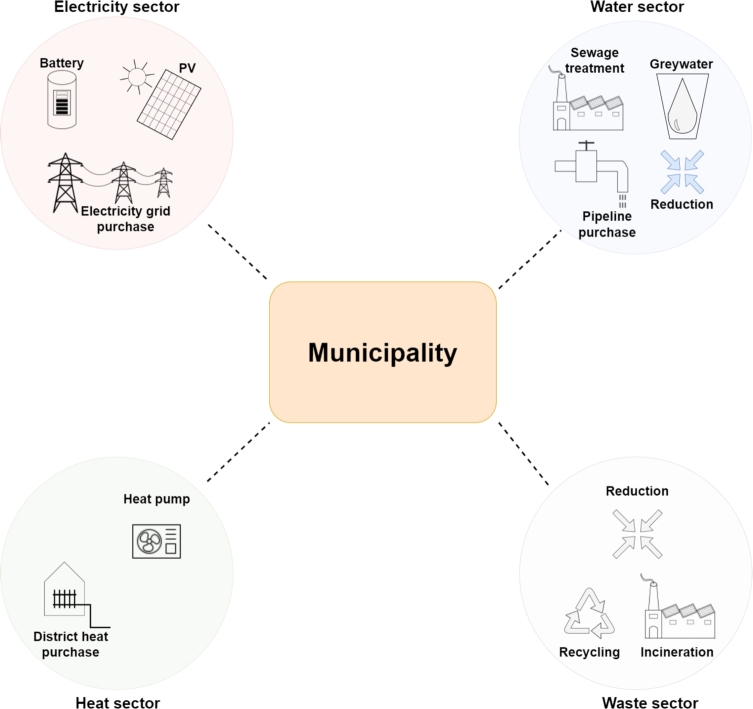
Case study setup for municipality analyses.

The analyses are performed in the municipality “Breitenau am Steinfeld” [101], Lower Austria, consisting of 730 households that are aggregated. Fig. 9 presents the technologies and operations considered in the municipality. Compared to the community, municipal analyses additionally consider recovered water from sewage treatment and recovered electricity and heat from waste incineration.

The analyses are similar to community analyses, with a significant difference in technology potential and demand scope. Another important change compared with the community is that investment decisions in sewage treatment plants and waste incineration plants are performed instead of resource disposal. Recovered water from sewage treatment and recovered electricity and heat from waste incineration can be used within the municipality and can contribute to SDG target achievement.

#### 4.3.2 Politically driven goal achievement in municipalities

This section presents the results of the SDG contribution target sensitivity analyses (Path 1) for the municipality. Detailed results of the simultaneous SDG sensitivity analyses are presented in the Appendix.

**Figure 10:**
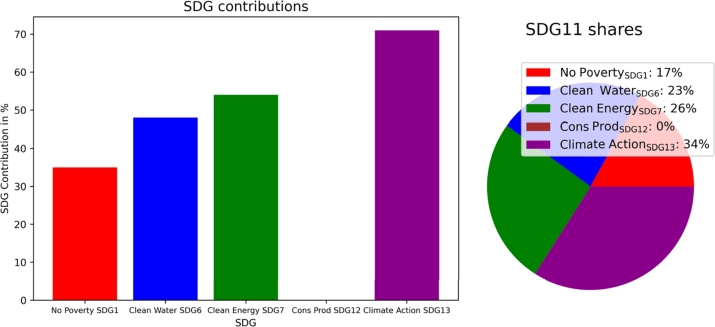
Municipality contribution to SDGs (left) and share of single SDG contributions to SDG11 (sustainable cities and communities) (right).

Fig. 10 shows that unlike for the municipality, SDG6 contribution is at  without constraints due to recovered water from sewage treatment. SDG7 is at , SDG13 is at  and SDG12 is not targeted without constraints. High cost reductions can be achieved by utilizing recovered electricity and heat from waste incineration. However, the non-biogenic share of waste limits SDG7 target achievement, as in the municipal SDG7 indicator denominator, the total share of recovered energy from waste incineration (biogenic and non-biogenic) is considered.

**Figure 11:**
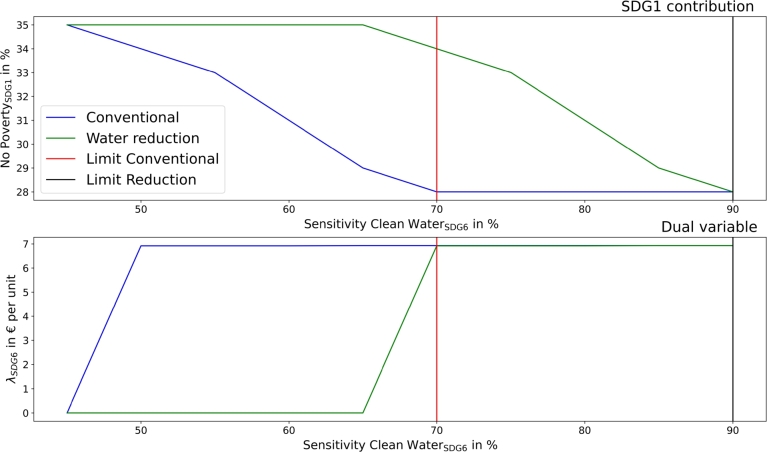
SDG1 - no poverty (top) and the dual variable of SDG6 (clean water and sanitation) in dependency of SDG6 contribution targets in the municipality.

Fig. 11 presents the sensitivity analysis for SDG6 (clean water and sanitation) in the municipality.

The limit for SDG6 is at  and can be improved to  with additional water reduction. The dual variables increase to a constant value when greywater is needed at targets of  and an almost linear cost increase emerges with additional greywater requirements.

**Figure 12:**
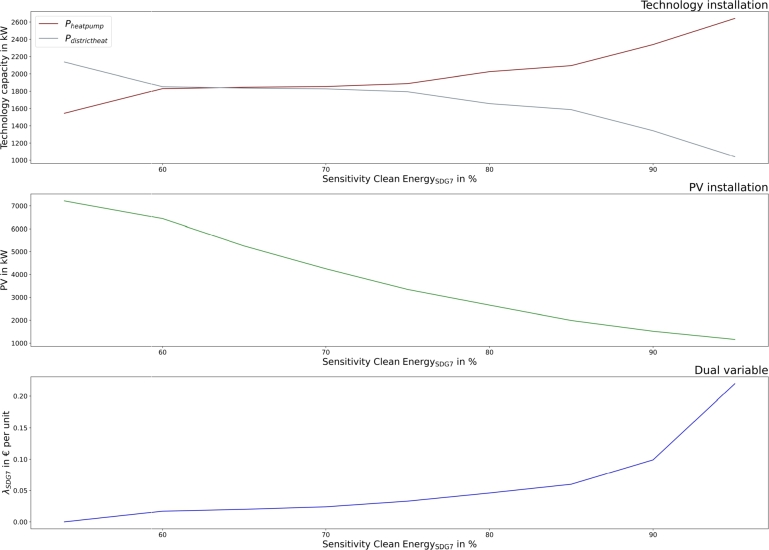
Municipality heat technology installation (top) and the dual variable (bottom) of SDG7 (clean and affordable energy) in dependency of SDG7 contribution targets.

Similar to the community, heat pump installation increases with higher goals for SDG7 (clean and affordable energy) and batteries are installed for higher local clean energy use. Moreover, PV installation decreases at higher target settings to prevent electricity feed-in, as owing to waste incineration energy recovery, an already high amount of excess electricity exists in the municipality. Fig. 12 presents the SDG7 sensitivity analysis.

**Figure 13:**
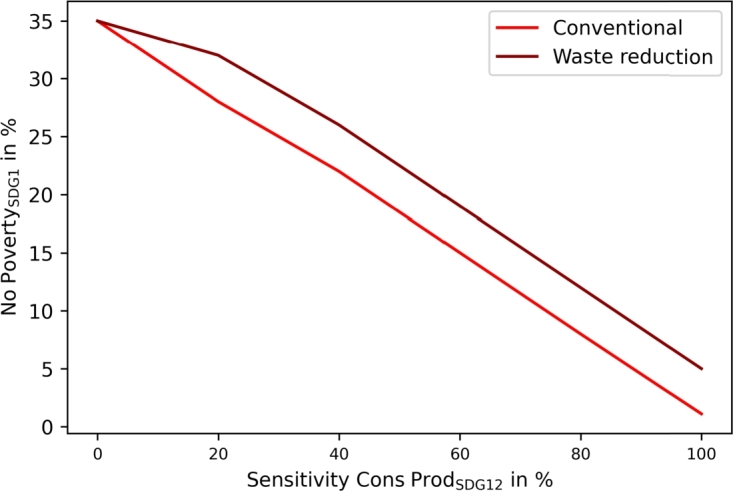
Municipality sensitivity analysis for SDG12 (responsible consumption and production) with and without waste reduction.

Fig. 13 presents the sensitivity analysis on SDG12 (responsible consumption and production), where similar increasing costs with increasing waste recycling can be examined.

**Figure 14:**
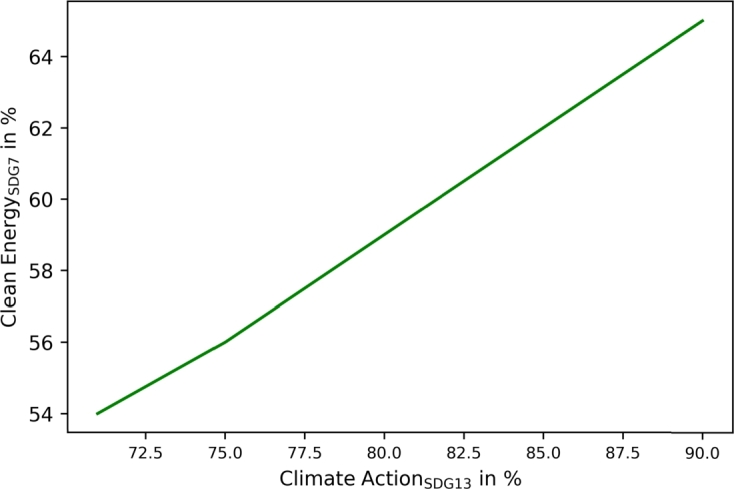
Correlation between SDG13 (climate action) and SDG7 (clean and affordable energy) in municipalities.

SDG13 (climate action) and SDG7 correlate linearly, as presented in Fig. 14. Unlike in communities, no saturation emerges.

The KKT constraints can identify active SDG constraints in the municipality. Similar to the community, SDG12 becomes active at  and SDG6 becomes active at  because of the implemented sewage treatment water recovery. SDG7 becomes active at , followed by SDG13 at . SDG6 reaches the limit at ; SDG13 at ; and SDG7 at . SDG12 is not limited because of the assumption of unlimited recycling programs.

#### 4.3.3 Politically driven incentive schemes in municipalities

This section presents the results of municipal incentive schemes, whereas detailed analyses are presented in the Appendix. Similar to the community analyses, both incentive schemes lead to the same results as SDG target policies.

Table 5 presents the applied incentive schemes in the municipality.

**Table 5:** Comparison of policy paths 2 and 3 regarding incentive cost volume, total community costs and cost increases in the municipality.

Policy	Incentive	Incentive costs in k€	Total community costs in k€	Cost increase in %
No incentives	-	0	1047	0
Sewage treatment penalty		244	1169	11.60
Greywater incentive		134	1169	11.64
District heat procurement penalty		28	1084	3.50
Heat pump subsidies		126	1068	1.98
Waste disposal penalties		0	1592	52.00
Waste recycling subsidies		554	1592	52.00
CO_2_ price	_CO2_	594	1476	40.97
CO_2_ price	_CO2_	55	1054	0.65
Combination penalties	-	294	1645	57.10
Combination subsidies	-	715	1668	59.30
Combination half subsidies, half penalties	-	154	1600	52.70

For single SDG investment schemes, penalties and subsidies have a similar relation as in communities. However, for multiple SDG incentives, the allocation changes, as subsidies lead to higher incentive costs than penalties. As in the previous analysis of communities, combining both incentive schemes results in the lowest costs.

## 5 Discussion

This section discusses the significant outcomes of the case study analyses. Section 5.1 provides a discussion on the applicability and scope of the proposed indicator system. Section 5.2 compares different SDG policy paths and the proposed incentive schemes.

### 5.1 Application of the proposed UN SDG classification system

The results in Sections 4.2 and 4.3 showed that the application of the SDG indicator system differs between particular SDG to be achieved. Efficient operation and technology installation can influence energy- and resource-related SDGs. However, policymakers must incentivize communities and municipalities to invest in sustainable development. SDG1 (no poverty) and SDG13 (climate action) require comparison setups. In the proposed method, business-as-usual configurations were provided as benchmarks. However, for long-term applicability, it might be more efficient to give community and municipality benchmarks with broader applicability. This can include comparison with communities and municipalities of similar scope, particularly providing an efficiency standard for communities and municipalities as business-as-usual.

The proposed SDG indicators differ slightly between communities and municipalities, leading to more sustainable operations in both. The application of SDG7 (clean and affordable energy) targets automatically leads to SDG13 improvement in both, as presented in Figure 8, Figure 14. However, the results showed different implementations and achievable goals in both configurations, as communities and municipalities differ in scope. Communities can improve SDG6 (clean water and sanitation) by greywater system installation. These are not cost-efficient, and would only be installed with the application of SDG6 targets. SDG7 can be improved in communities by decarbonizing heat generation. Municipality analyses introduce water recovery as a direct contribution to SDG6, and waste energy recovery, which SDG7 assessed. The introduction of waste energy recovery leads to a simultaneous improvement and limitation in SDG7 contribution. Recovered water only improves SDG6 and has no counter-effect. Furthermore, Figure 5, Figure 7, Figure 11, Figure 13 show that both, the community and municipality should apply waste and water reduction. This can increase SDG6 and SDG12 (responsible consumption and production) improvement while keeping SDG1 at a higher level.

The application of the proposed indicators requires critical implementation assessment. Fig. 12 shows that SDG target setting could also affect sustainable technology installation, as PV installation is decreased with higher SDG7 targets in the municipality. Therefore, a critical assessment of the SDG indicator applicability in specific setups might be necessary to avoid counter-effects. This assessment is the responsibility of municipal governments and policymakers, who establish the SDG indicator system. Municipality actions can include cooperation with large electricity consumers or flexibilities that can use the excess electricity. Policy actions can establish an indicator adaptation for the proposed system, where fed-in electricity is not counted or less counted for the indicator. However, a general indicator adaption is not purposeful, as the impact might be completely different in other configurations with less excess electricity. Therefore, if an indicator adaption for the municipality is desired, justification must be made by the municipal government.

### 5.2 Comparison of policy paths

The method established three different SDG policy paths for the improvement of sustainable development in the community and municipality. The proposed SDG policy paths introduced in Fig. 2 all lead to desired SDG contribution targets, whereas the implementation of the paths differs in their applied policy actions. In terms of goal achievement, all three paths lead to the desired SDG goals, but they differ significantly in costs incurred for target achievement. Proposing strict SDG targets in Path 1 increases costs, especially for greywater installation. Therefore, the SDG1 indicator is mandatory for policymakers to have an overview of community or municipality costs. Moreover, the dual variables of the constraints in Path 1, representing the SDG targets, are an efficient means to determine costs for SDG achievement. They can give an insight until which goals the costs do not increase disproportionally high, as it can be seen in Fig. 8. These dual variables can therefore help in defining penalties for policy Path 2 as they represent the costs for higher SDG target achievements. The results showed that when the operation that lowers the SDG contribution can be identified, dual variables as penalties lead to the desired goal achievement. However, penalties lead to a direct cost increase for consumers in the community or municipality as these penalties must be directly paid by the consumers. Path 3 can provide an alternative, as funding agencies can support communities and municipalities in higher goal achievement through technology investment subsidies. The technologies, that must be subsidized can be identified in Path 2 by analyzing which technologies are increasingly installed to avoid penalty costs. Path 3 also leads to the desired goals, but it leads to a high subsidy load for funding agencies, which consumers indirectly pay in the form of taxes. Thus, comparing incentive schemes according to Paths 2 and 3, as presented in Table 4, Table 5 is mandatory for policymakers to define such schemes and to identify to lowest costs incurred for higher SDG targets.

Paths 2 and 3 differ in their impact on the incentive scheme costs, depending on the desired SDG contribution targets. SDG6 contribution in the form of greywater installation is not performed without policy incentives. Sewage treatment penalties lead to higher costs than greywater investment subsidies. Therefore, investment subsidies should be prioritized for SDG6. Regarding SDG7, penalties on district heat procurement are more cost-efficient than heat pump investment subsidies. District heat penalties are equivalent to increasing market prices. Thus, incentive schemes to promote heat pumps might not be necessary for the long-term if district heat prices increase because of changes in the market. SDG12 can be improved by either recycling promotion or disposal penalizing. However, recycling promotion might need more information on recycling programs, whereas disposal penalizing might encourage consumers to look for alternative options to disposal, which can include participation in recycling programs. Higher CO_2_ prices lead to higher SDG13 contribution. However, SDG13 is also automatically improved with incentive schemes for SDG7. The results showed that SDG7 incentive schemes are more cost-efficient than SDG13 penalties while leading to similar SDG13 contributions. Thus, the promotion of clean energy technologies should be favored to emission penalizing. Multiple target achievement incentive schemes vary between communities and municipalities, as presented in Table 4 and Table 5. Thus, a scope-dependency of the incentive schemes could be identified. If only one kind of incentive scheme is established, investment subsidies should be favored in communities, whereas penalties should be favored in municipalities. However, the results for both, community and municipality, showed that a combination of penalties and investment subsidies leads to the lowest incentive costs while reaching similar SDG contribution targets. Funding agencies can use penalties to finance at least a share of the provided investment subsidies. Penalties or investment subsidies for single SDG targets should be chosen based on the higher cost-efficiency of the respective incentive scheme. Therefore, the incentive scheme's definition must depend on the particular SDG.

## 6 Conclusions

This work introduces an SDG indicator system that indicates communities' or municipalities' contributions to the energy- and resource-related SDGs. The method applied the proposed indicators in communities and municipalities by simulating both with linear optimization models. Application leads to differences in technology impact, SDG target costs and SDG target limits. Furthermore, technology portfolios and investments have a high impact on SDG target achievement. The heat sector greatly affects the indicators, especially SDG7 and SDG13 (climate action). Increasing heat pump installation in favor of district heat connection has positive contributions to SDG7 and SDG13 while leading to only slightly increasing costs. However, sustainable water management, represented by SDG6 (clean water and sanitation), leads to high costs for the community or municipality. The main option for higher goal achievement is a greywater system installation, which leads to high investment costs. Thus, SDG1 (no poverty) must be considered in the indicator system to monitor the financial load of SDG contribution improvement for consumers. Therefore, policymakers establishing indicator systems for sustainable technology utilization must consider all desired SDGs and the required costs for achievement.

The model considers three different policy paths, namely, SDG target setting (Path 1), penalty charging (Path 2), and investment subsidies (Path 3). Dual variables of Path 1 constraints are considered penalties for counterproductive actions in Path 2. Therefore, dual variables are applicable to policy action settings. Moreover, the approach identifies technologies that must be promoted for sustainable development and must be subsidized in Path 3. However, the costs for consumers to achieve targets differ between paths. For specific SDGs, penalty setting leads to lower costs, while for other SDGs, investment subsidy provision is more cost-efficient. However, combining both incentive schemes leads to the lowest incentive costs. The decision on penalty or subsidy must be performed concerning the single SDGs.

This work considers SDGs and policy impact on communities and municipalities within their system boundaries. However, the overall impact of the implementation on the economy and society beyond the system boundaries is not considered. Positive or negative effects on communities' and municipalities' SDG target achievements can also depend on external influences. As SDGs are an international issue, the impact of multiple communities and municipalities implementing such measuring systems on national or global SDG contribution should be further assessed. Furthermore, it should be assessed how technologies that are out of the scope of the communities and municipalities can contribute to SDG goal contribution. Moreover, additional technologies from other sectors, such as hydrogen technologies, can be implemented within or beyond the system boundaries. Therefore, future work should consider the interaction between multiple communities and municipalities, implementing classification systems also considering effects beyond their system boundaries.

## Nomenclature

**Table 6:** Model parameters and decision variables.

**Sets**		
SDG	Energy- and resource-related SDG	index: k
T	Available technologies	index: l
**Parameters**		
*WACC*	Weighted average cost of capital	%
*N*_*l*_	Amortization period for technologies	-
$C_{l}^{\mathrm{invest}}$	Capacity-based costs	€/[l]
*D*_*water*,*com*_	Water demand community	
*D*_*water*,*mun*_	Water demand municipality	
$F_{elgrid}^{\mathrm{ren}}$	Share renewables electricity grid	-
$F_{dhgrid}^{\mathrm{ren}}$	Share renewables district heat grid	-
$F_{waste}^{\mathrm{biogene}}$	Share biogenic waste	-
$M_{waste}^{\mathrm{total}}$	Total accruing waste	
$TG_{com}^{\mathrm{cleanwate}r_{\mathrm{SDG6}}}$	SDG6 target community	-
$TG_{mun}^{\mathrm{cleanwate}r_{\mathrm{SDG6}}}$	SDG6 target municipality	-
$TG_{com}^{\mathrm{cleanenerg}y_{\mathrm{SDG7}}}$	SDG7 target community	-
$TG_{mun}^{\mathrm{cleanenerg}y_{\mathrm{SDG7}}}$	SDG7 target municipality	-
$TG^{\mathrm{conspro}d_{\mathrm{SDG12}}}$	SDG12 target	-
$TG^{\mathrm{climateactio}n_{\mathrm{SDG13}}}$	SDG13 target	-
**Variables**		
*z*	Objective	€
*c*^tot^	Total costs	€
*c*^tot,BaU^	Total costs business-as-usual scenario	€
*c*^invest^	Investment costs	€
*c*^operational^	Operational costs	€
*c*^procurement^	Procurement costs	€
*α*	Annuity factor	-
*x*_*l*_	Capacity investment	[*l*]
$v_{water,com}^{\mathrm{reduced}}$	Reduced water community	
$v_{water,com}^{\mathrm{greywater}}$	Reused greywater community	
$v_{water,mun}^{\mathrm{reduced}}$	Reduced water municipality	
$v_{water,mun}^{\mathrm{greywater}}$	Reused greywater municipality	
$v_{water,mun}^{\mathrm{recovered}}$	Recovered water municipality	
$q_{el,com}^{\mathrm{PV}}$	PV generation community	
$q_{el,com}^{\mathrm{elgrid}}$	Electricity grid procurement community	
$q_{heat,com}^{\mathrm{HP}}$	Heat pump heat generation community	
$q_{heat,com}^{\mathrm{dhgrid}}$	District heat grid procurement community	
$q_{el,com}^{\mathrm{feedin}}$	Electricity grid feed-in community	
$q_{el,mun}^{\mathrm{PV}}$	PV generation municipality	
$q_{el,mun}^{\mathrm{elgrid}}$	Electricity grid procurement municipality	
$q_{heat,mun}^{\mathrm{HP}}$	Heat pump heat generation municipality	
$q_{heat,mun}^{\mathrm{dhgrid}}$	District heat grid procurement municipality	
$q_{el,mun}^{\mathrm{feedin}}$	Electricity grid feed-in municipality	
$q_{el,mun}^{\mathrm{wastecomb}}$	Waste treatment recovered electricity municipality	
$q_{heat,mun}^{\mathrm{wastecomb}}$	Waste treatment recovered heat municipality	
$q_{heat,mun}^{\mathrm{exhaust}}$	Exhaust heat municipality	
$m_{waste}^{\mathrm{recycled}}$	Recycled waste	
$m_{waste}^{\mathrm{reduced}}$	Reduced waste	
*em*^tot^	Total CO_2_ emissions	_CO2_
*em*^tot,BaU^	CO_2_ emissions business-as-usual	_CO2_
$q_{el,com}^{\mathrm{ren}}$	Renewable electricity community	
$q_{el,com}^{\mathrm{tot}}$	Total electricity community	
$q_{el,mun}^{\mathrm{ren}}$	Renewable electricity municipality	
$q_{el,mun}^{\mathrm{tot}}$	Total electricity municipality	
$i^{\mathrm{nopovert}y_{\mathrm{SDG1}}}$	SDG1 indicator	-
$i_{com}^{\mathrm{cleanwate}r_{\mathrm{SDG6}}}$	SDG6 indicator community	-
$i_{mun}^{\mathrm{cleanwate}r_{\mathrm{SDG6}}}$	SDG6 indicator municipality	-
$i_{com}^{\mathrm{cleanenerg}y_{\mathrm{SDG7}}}$	SDG7 indicator community	-
$i_{mun}^{\mathrm{cleanenerg}y_{\mathrm{SDG7}}}$	SDG7 indicator municipality	-
$i^{\mathrm{conspro}d_{\mathrm{SDG12}}}$	SDG12 indicator	-
$i^{\mathrm{climateactio}n_{\mathrm{SDG13}}}$	SDG13 indicator	-
$i_{old}^{\mathrm{SDG},k}$	Nonweighted SDG indicator	-
$i_{new}^{\mathrm{SDG},k}$	Weighted SDG indicator	-
$\lambda_{com}^{\mathrm{cleanwate}r_{\mathrm{SDG6}}}$	SDG6 dual variable community	€/[*goal*]
$\lambda_{mun}^{\mathrm{cleanwate}r_{\mathrm{SDG6}}}$	SDG6 dual variable municipality	€/[*goal*]
$\lambda_{com}^{\mathrm{cleanenerg}y_{\mathrm{SDG7}}}$	SDG7 dual variable community	€/[*goal*]
$\lambda_{mun}^{\mathrm{cleanenerg}y_{\mathrm{SDG7}}}$	SDG7 dual variable municipality	€/[*goal*]
$\lambda^{\mathrm{conspro}d_{\mathrm{SDG12}}}$	SDG12 dual variable	€/[*goal*]
$\lambda^{\mathrm{climateactio}n_{\mathrm{SDG13}}}$	SDG13 dual variable	€/[*goal*]

## CRediT authorship contribution statement

Matthias Maldet: Conceived and designed the experiments; Performed the experiments; Analyzed and interpreted the data; Contributed reagents, materials, analysis tools or data; Wrote the paper.

Georg Lettner: Analyzed and interpreted the data; Contributed reagents, materials, analysis tools or data.

Christoph Loschan; Daniel Schwabeneder; Hans Auer: Conceived and designed the experiments; Analyzed and interpreted the data

## Declaration of Competing Interest

The authors declare that they have no known competing financial interests or personal relationships that could have appeared to influence the work reported in this paper.

## Figures and Tables

**Table A.7:** Tariff assumptions.

Tariff	Value
Electricity purchase	
Electricity feedin	
District heat purchase	
Water pipeline purchase	
Recovered water purchase	
Waste disposal costs	
Waste recycling costs [103]	
Sewage disposal costs	
CO_2_ price	

**Table A.8:** Investment cost assumptions with a WACC of .

Technology	Costs	Amortization period in years [104]	Source
PV		20	[105]
Battery		10	[106]
Heat pump		8	[107], [108]
District heat connection		20	[109]
Waste combustion		10	[110]
Sewage treatment plant		20	[111], [112]
Greywater system	-	15	[113]

**Table A.9:** Technology capacity assumptions in the community and municipality.

Technology	Community	Municipality
PV		
Battery		
Heat pump	_therm_	_therm_
District heat connection		
Waste combustion	-	
Sewage treatment plant	-	
Greywater system		

**Table A.10:** Annual energy and resource demands in community and municipality.

Sector	Community	Municipality	Sources
Electricity			[114], [115]
Heat			[116], [117]
Water			[118]
Waste			[119]

**Table A.11:** Emission assumptions for technologies and actions.

Technology	Emissions	Source
Electricity grid	_CO2_/kW h	[120]
District heat	_CO2_/kW h	[121]
Sewage disposal	_CO2_/m^3^	[122]
Waste disposal	_CO2_/kg	[123]
Sewage treatment	_CO2_/m^3^	[122]
Waste combustion	_CO2_/kg	[123]

**Table B.1:** Community technology installation impact of SDG Path 1.

Sens. in %	PV in kWp	Battery in kW h	Heat pump in kW	District heat in kW	Grey water systems in l
0	30.00	3.65	3.93	13.86	0
5	30.00	3.65	3.93	13.86	8
45	30.00	3.65	3.93	13.86	150
50	30.00	3.65	4.69	13.10	150
70	30.00	3.95	14.47	3.32	150
90	29.86	3.98	14.21	3.58	150
100	30.00	4.00	14.97	2.80	150

**Table B.2:** Community SDG contribution target impact SDG Path 1.

Sens. in %	SDG1	SDG6	SDG7	SDG12	SDG13
0	21	0	73	0	42
5	20	5	73	5	42
45	-4	45	73	45	47
50	-5	45	75	50	50
70	-10	45	90	88	70
90	-11	45	90	90	70
100	-11	45	90	100	70

**Table B.3:** Community technology installation impact SDG Path 2.

Policy action	PV in kWp	Battery in kW h	Heat pump in kW	District heat in kW	Grey water systems in l
Sewage disposal penalties	30	3.65	3.93	13.86	67
District heat penalties	30	3.65	10.50	7.31	0
Waste disposal penalties	30	3.65	3.93	13.86	0
CO_2_ price extension	30	3.95	14.47	3.32	0
Penalty combination	30	3.68	11.22	6.57	67

**Table B.4:** Community SDG contribution target impact of SDG Path 2.

Policy action	SDG1 in %	SDG6 in %	SDG7 in %	SDG12 in %	SDG13 in %
Sewage disposal penalties	-29	29	73	0	43
District heat penalties	19	0	87	0	57
Waste disposal penalties	12	0	73	100	51
CO_2_ price extension	-20	0	90	50	65
Penalty combination	-42	29	88	100	68

**Table B.5:** Community technology installation impact of SDG Path 3.

Policy action	PV in kW p	Battery in kW h	Heat pump in kW	District heat in kW	Grey water systems in l
Greywater investment subsidies	30	3.65	3.93	13.86	60
Heat pump investment subsidies	30	3.65	8.88	8.90	0
Waste recycling subsidies	30	3.65	3.93	13.86	0
CO_2_ price extension	30	3.68	6.57	11.20	0
Subsidy combination	30	3.68	110.81	6.97	60

**Table B.6:** Community SDG contribution target impact of SDG Path 3.

Policy action	SDG1 in %	SDG6 in %	SDG7 in %	SDG12 in %	SDG13 in %
Greywater investment subsidies	22	28	73	0	42
Heat pump investment subsidies	23	0	85	0	55
Waste disposal penalties	22	0	73	100	51
CO_2_ price extension	18	0	80	0	50
Subsidy combination	22	28	87	100	68

**Figure B.1:**
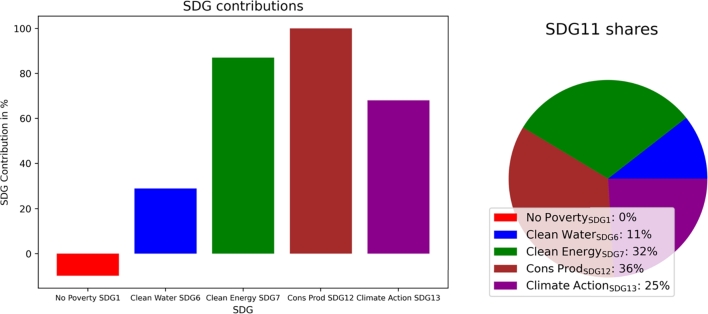
Combined incentive scheme for communities.

**Table B.7:** Municipality technology installation impact SDG Path 1.

Sens. in %	PV in kWp	Bat in kW h	HP in kW	DH in kW	GW in l	Inc. in kW	Sew. treat in l
0	7214	634	1545	2137	0	579	8025
5	7214	638	1550	2132	0	550	8025
50	7214	754	1588	2094	420	283	7600
60	7195	817	1877	1805	2110	241	5900
70	5098	790	1934	1748	3800	239	4220
85	3172	779	2538	1144	3800	156	4220
90	3993	2663	3311	371	4012	118	4012
95	4005	6459	3444	238	4012	66	4012
100	4044	6639	3457	225	4012	0	4012

**Table B.8:** Municipality SDG contribution target impact SDG Path 1.

Sens. in %	SDG1 in %	SDG6 in %	SDG7 in %	SDG12 in %	SDG13 in %
0	35	48	54	0	71
5	33	48	54	5	71
50	18	50	56	50	74
60	13	60	60	60	80
70	6	70	70	70	81
85	-4	70	85	85	85
90	-29	70	90	90	90
95	-65	70	95	95	90
100	-68	70	95	100	90

**Table B.9:** Municipality technology installation impact of SDG Path 2.

Policy action	PV in kWp	Bat. in kW h	HP in kW	DH in kW	GW in l	Inc. in kW	Sew. treat in l
Sew. treat pen	7214	677	1570	2112	4010	454	4012
DH pen.	7214	749	2455	1228	0	579	8025
Inc. pen.	7214	807	1537	2145	0	0	8025
CO_2_ price ext.	7214	1189	2952	730	4012	1489	4012
Pen. combi.	7214	880	2635	7047	4012	0	4012

**Table B.10:** Municipal SDG contribution target impact of SDG Path 2.

Policy action	SDG1 in %	SDG6 in %	SDG7 in %	SDG12 in %	SDG13
Sew. treat pen.	12	71	55	0	73
DH pen.	31	48	61	0	83
Inc. pen.	1	48	57	100	74
CO_2_ price ext.	-29	71	66	21	92
Pen. combi.	-20	71	66	100	89

**Table B.11:** Municipality technology installation impact of SDG Path 3.

Policy action	PV in kWp	Bat. in kW h	HP in kW	DH in kW	GW in l	Inc. in kW	Sewage treat in l
GW inv. sub.	7214	677	1570	2112	4012	454	4012
HP inv. sub.	7214	727	2211	1471	0	579	8025
Rec. sub.	7214	807	1537	2145	0	0	8025
CO_2_ price ext.	7214	744	1911	1771	0	579	8025
Sub. combi.	7214	880	2583	1099	4012	0	4012

**Table B.12:** Municipal SDG contribution target impact of SDG Path 3.

Policy action	SDG1	SDG6	SDG7	SDG12	SDG13
GW inv. sub.	71	55	0	73	36
HP inv. sub	48	60	0	81	42
Rec. sub.	48	57	100	74	36
CO_2_ price ext.	48	58	0	77	31
Sub. combi.	71	65	100	89	41

**Figure B.2:**
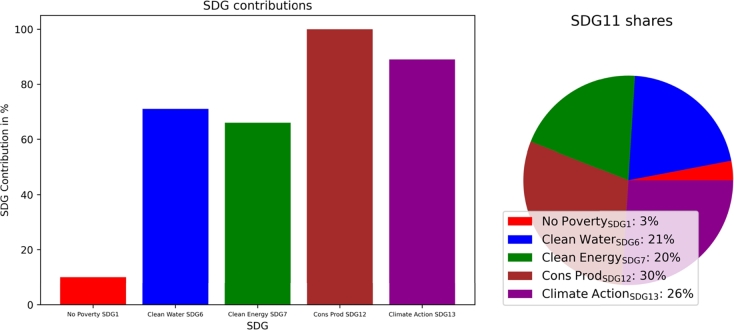
Combined incentive scheme for municipality.

## Data Availability

Data included in article/supp. material/referenced in article.

## Appendix A Data assumptions

This section provides an overview of the assumptions in the case studies presented in Sections 4.2 and 4.3.

The community and municipality's business-as-usual (BaU) values are determined by separate optimization analyses without sustainable operations and technology investment. BaU results for the community are 23766 € as total costs and _CO2_ for the emissions. For the municipality, BaU results indicate 2438 k€ and _CO2_.

Table A.7 presents the tariff assumptions for both, the community and municipality.

Table A.8 presents the technology investment cost assumptions. Operational costs are assumed as in [99].

Table A.9 presents the assumed maximum technologies in the community and municipality. Technologies were considered based on the case study sites' provided community and municipality data.

Table A.10 gives an overview on the energy and resource demands. Demands were assumed based on household sizes, whereas for the municipality, demands of public buildings were provided by the investigated municipality. Moreover, the total accruing waste amount was provided for the whole municipality.

Finally, Table A.11 presents the emission assumptions. Waste emissions are adapted, as only the nonbiogenic share of waste leads to emissions. However, sludge as a by-product from sewage treatment is also incinerated and counted as biogenic, leading to a higher total biogenic percentage and thus, to lower waste incineration emissions. Disposal and treatment of waste and sewage are assumed with the same emissions, as disposal leads to treatment beyond the system boundaries.

Moreover, the share of renewable energy generation in the electricity grid of  [124] and in the district heating grid of  [121] must be considered.

## Appendix B Case study results

This section summarizes the detailed results of the community and municipality analyses for all paths according to Fig. 2.

### B.1 Community case study results

This section focuses on the results of the policy paths in the community.

The following tables present the sensitivity analysis for specific SDG target setting in the community, according to Path 1. Table B.1 illustrates the impact on technology installation and Table B.2 shows the effects on SDG contribution targets. Only the relevant sensitivities where SDG targets become active or reach their limit are presented.

Heat pumps and greywater systems are increasingly installed to reach the proposed SDG targets. District heating connection is decreased, whereas other technologies are only slightly affected. Emission reductions are limited because of the high dependency on the electricity grid, a significant source of emissions in the community. SDG1 becomes negative at targets of . Thus, the costs are higher than that in the BaU case, leading to non-cost-efficient operation. However, the reduction of water and waste (as additional sensitivity) can extend the SDG limits and keeps SDG1 at a higher level while reaching the same targets.

Table B.3 presents the impact of penalty incentive schemes on technology installation in the community and Table B.4 presents their implications on SDG contribution targets. CO_2_ price extension considers prices of _CO2_. The exact values of the investment schemes are presented in Table 4.

Increased sewage disposal costs lead to greywater installation becoming an economically feasible option and thus to increased SDG6 contribution. Moreover, additional district heating costs lead to heat pumps becoming the dominant heat generation technology. Additional heat pump installation leads to improvement in SDG7 contribution. Increasing waste disposal costs lead to full waste recycling (as no limit was assumed), which leads to a maximum SDG12 target. High CO_2_ prices increase SDG13. Such prices affect not only SDG13 but also SDG7 and SDG12. However, all of the penalties lead to increased total costs and thus, to a decrease in SDG1. Incentive schemes that consider greywater further lead to negative SDG1 contributions and thus to higher costs than those in the BaU scenario. Therefore, a detailed analysis on the incentive costs (as performed in Table 4) is mandatory for penalty impact assessment.

Table B.5, Table B.6 present the investment subsidy incentive schemes and their impact on technology installation and SDG target achievement. CO_2_ price extension considers prices of _CO2_.

Investment subsidies lead to similar results as penalties. Therefore, investment subsidies can be an alternative approach to promote SDG contribution. SDG1 does not decrease, as the investment incentives are not directly provided by consumers. However, as it must be assumed that the funding agency provides the investment subsidies from tax revenues, investment incentives are also indirectly paid by consumers. Therefore, the comparison must be performed in terms of costs for incentive schemes.

A combination of penalties and investment subsidies also leads to similar results as those in Paths 2 and 3 in particular while keeping SDG1 at a higher level than that with penalties alone. Fig. B.1 presents the results of a combined incentive scheme approach in the community.

In summary, all three policy paths in the community lead to similar results. However, Path 1 requires strict policy setting, whereas Paths 2 and 3 require incentive scheme costs. Moreover, combining penalties and investment subsidies leads to the most efficient incentive scheme in the community.

### B.2 Municipality case study results

This section focuses on the results of the policy paths in the municipality.

Table B.7, Table B.8 present the impact of the SDG target sensitivity analyses on technology installation and SDG contribution according to policy Path 1. Table B.7 includes PV, battery (Bat), heat pump (HP), district heat (DH), greywater (GW), waste incineration (Inc.) and sewage treatment (Sew. treat) capacities.

As for the community, heat pump installation increases in favor of district heat installation. Moreover, PV installation decreases to prevent electricity excess feed-in electricity. Owing to the implemented waste incineration energy recovery, the amount of excess electricity in the system is already high without PV installation. Thus, additional PV installation leads to increased SDG7 contribution in the system. SDG6 can be achieved without greywater installation due to the implemented sewage treatment water recovery, which is the significant difference to the community analyses. With the implemented water reduction, the limit of SDG7 can be extended to .

The Table B.9, Table B.10 show the penalty (pen) incentive scheme impact of Path 2. Exact investment scheme values are presented in Table 5. Extended CO_2_ prices are assumed at _CO2_.

As in the community, sewage treatment penalties increase greywater system installation. Moreover, more expensive district heat procurement costs promote heat pump establishment and waste combustion penalties encourage recycling. However, high CO_2_ prices and greywater installation lead to higher costs than in the BaU scenario. Therefore, similar to the community, detailed investment scheme costs are mandatory to analyze.

Finally, Table B.11 shows the impact of municipal investment subsidy (inv. sub.) incentive schemes on technology installation. Table B.12 presents the impact on SDG target contributions. Both tables show the results according to municipal policy Path 3. Extended CO_2_ prices are assumed at _CO2_.

The proposed investment subsidies lead to similar results as those of penalties, with the difference that the subsidies are indirectly covered by municipal consumers by tax payments. Therefore, SDG1 is not the only indicator to be considered. Incentive scheme costs are mandatory to be considered as well.

Similar to the community, a combination of penalty and investment subsidy incentive schemes, particularly a combination of Paths 2 and 3, leads to the lowest costs and is therefore the most efficient incentive scheme. Fig. B.2 presents the SDG contributions of a combined incentive scheme.
